# Socioeconomic inequality in exclusive breastfeeding behavior and ideation factors for social behavioral change in three north-western Nigerian states: a cross-sectional study

**DOI:** 10.1186/s12939-021-01504-4

**Published:** 2021-07-27

**Authors:** Dele Abegunde, Paul Hutchinson, Udochisom Anaba, Foyeke Oyedokun-Adebagbo, Emily White Johansson, Bamikale Feyisetan, Emma Mtiro

**Affiliations:** 1 Breakthrough RESEARCH/Nigeria, Plot 839 Idris Ibrahim Crescent Jabi, Abuja, Nigeria; 2 Population Council, Washington, DC USA; 3 Department of Global Community Health and Behavioral Sciences, School of Public Health and Tropical Medicine, Tulane University, New Orleans, Louisiana USA; 4 United States Agency for International Development (USAID), Abuja, Nigeria

**Keywords:** Inequality, Exclusive breastfeeding practice, Social and behavioral change communication, Nigeria

## Abstract

**Background:**

Socioeconomic inequalities could mitigate the impact of social and behavior change (SBC) interventions aimed at improving positive ideation towards the practice of exclusive breastfeeding. This study explores the empirical evidence of inequalities in the practice of exclusive breastfeeding (EBF) and associated ideational dimensions and domains of the theory of Strategic Communication and Behavior Change in three north-western Nigeria states.

**Methods:**

We used cross-sectional data from 3007 randomly selected women with under-two-year-old children; the convenient regression method was applied to estimate the concentration indexes (CIxs) of exclusive breastfeeding behavior, ranked by household wealth index. Inequality was decomposed to associated ideational factors and sociodemographic determinants. Avoidable inequalities and the proportion of linear redistribution to achieve zero inequality were estimated.

**Results:**

Women from wealthier households were more likely to practice exclusive breastfeeding CIx = 0.1236, *p*-value **=** 0.00). Attendance of at least four antenatal clinic visits (ANC 4+) was the most significant contributor to the inequality, contributing CIx = 0.0307 (*p*-value = 0.00) to the estimated inequality in exclusive breastfeeding practice. The elasticity of exclusive breastfeeding behavior with respect to partners influencing decision to breastfeed and ANC4+, were 0.1484 (*p*-value = 0.00) and 0.0825 (*p*-value = 0.00) respectively. Inequality in the regular attendance at community meetings (CIx = 0.1887, *p*-value =0.00); ANC 4+) (CIx = 0.3722, p-value = 0.00); and maternal age (CIx = 0.0161, p-value = 0.00) were pro-rich. A 10.7% redistribution of exclusive breastfeeding behavior from the wealthier half to the poorer half of the population could eliminate the inequality (line of zero inequality). Inequalities were mainly in the cognitive and social norms dimension and were all pro-poor.

**Conclusion:**

Socioeconomic inequalities exist in exclusive breastfeeding behaviors and in associated ideation factors in the three states but are mostly avoidable. A 10.7% redistribution from wealthier to the poorer half of the population will achieve elimination. Messaging for SBC communication interventions to improve breastfeeding practices could be more effective by targeting the mitigation of these inequalities.

**Table:** 

This article is a part of the Interventions and policy approaches to promote equity in breastfeeding collection, guest-edited by Rafael Pérez-Escamilla, PhD and Mireya Vilar-Compte, PhD	

## Background

Health inequities are significant determinants of population health, and interventions to improve population health, which neglect the impact of inequality on population health may in the long run, aggravate inequitable access to health, healthcare, and social injustice [1]. Health inequalities defined as the disproportionate concentration of individuals with certain health behaviors or outcomes in some population sub-groups, and the inequality in the delivery of healthcare remain challenges to the health policy community. The unequal exposure to interventions and access to health services generally give rise to inequitable and avoidable differences in disease burden and response to interventions to mitigate the burdens across groups in the population.

It is well recognized that social determinants shape individual interaction and play a significant role in the wellbeing of the individual, family, and community [2–4]. Factors such as knowledge, attitudes, social, mental, and cultural norms and conventions influence individuals’ and communities’ health states, including healthcare-seeking behaviors [5]. Globally, social and behavior change (SBC) interventions are being increasingly implemented for modulating change toward generating appropriate healthcare demand and reducing preventable maternal, newborn, and childhood morbidities and mortalities [6–8]. For instance, the United States Agency for International Development (USAID) is investing in SBC interventional research and programming globally and in Nigeria, aiming towards positively changing the norms that underpin the health-seeking behaviors of individuals, families and communities [9–11]. These SBC interventions are mainly social, and behavior change communication (SBCC) approaches and tools informed by behavioral theories addressing the barriers that prevent the practicing of lifesaving behaviors to improve health outcomes. There is limited but growing evidence on the nature of the interactions between social and cultural norms in modulating health-seeking behavior. Evidence is scarce on the inequities in the population distribution of health-improving priority behaviors and their underlying norms and ideations. The impact of interventions could be limited or skewed unfairly towards the relatively socioeconomically endowed group in the population. The existence of inequalities in health and health care, the priority behaviors that impact population health, and the ideational determinants could regressively impact SBC for demand creation programming. This situation could consequently result in suboptimal impact from SBC interventions diminishing the expected program outcomes.

Despite decades of heightened advocacy for improving breastfeeding within Nigeria Primary Health Care, breastfeeding practices remain low in Nigeria, with exclusive breastfeeding rates as low as 29% at the national level and as low as 19% in the country’s north-western region [12]. Studies have explored the social determinants of exclusive breastfeeding practices in diverse cultures. In northern Nigeria, sociodemographic factors such as maternal education, maternal employment, household wealth, antenatal care attendance, and facility delivery have been associated with breastfeeding practices [13, 14]. Other studies have also shown that cultural beliefs and perceptions about breastfeeding, including husband disapproval [15], knowledge, norms, self-efficacy, and other psychosocial influences, have also influenced breastfeeding practices [16, 17].

New strategies for improving breastfeeding practices and behavior have built on the Ideation Model of Strategic Communication and Behavior Change [16] theories, which groups individual ideation with behavior within three: cognitive, emotional, and social ideational dimensions as informed in behavioral theories and models [18]. This model incorporates constructs derived from several behavioral theories and models [ref] and allows the identification psychosocial factors that predict breastfeeding behavior and causal attribution of breastfeeding behavior change to communication interventions. Behavior-specific ideational factors combine to exert a cumulative influence on individual such that individuals with more enabling ideational factors are more likely to adopt or sustain a given behavior compared to those with less. An implicit assumption is that the probability of behavior change is higher with more positive ideation factors in an individual than those with less. Each dimension consists of domain of specific behavioral influences within it, including knowledge, attitudes, beliefs, perceived risk, subjective norms and self-image (in the cognitive dimension); emotional response, empathy, and self-efficacy (in the emotional dimension); and social support, social influence, interpersonal communication and personal advocacy (in the social dimension) [18] [19]. Social and Behavioral Change intervention programs typically focus on modulating psychosocial influences, or ideations, as intermediate determinants of health behaviors [16].

Notwithstanding the programming attention to the psychosocial determinants for improving breastfeeding practices, critical explorations are also essential for determining the extent to which inequalities in ideational norms shape behavior towards demand creation for Maternal, Newborn and Child Health and Nutrition (MNCH+N), Family Planning (FP), and Malaria care in the population. Evidence is scarce in the published literature on the causal determinants (predictors) of observed inequalities in a population’s breastfeeding behavior and practices and how existing inequalities could mitigate outcomes from SBC interventions and programming in communities. A study in rural India has explored the Effects of health behavior change intervention through women’s self-help groups on maternal and newborn health practices and related inequalities [20]. Another study from Norway explained the socioeconomic inequalities in exclusive breastfeeding. It concluded that socioeconomic inequalities in exclusive breastfeeding were “largely explained by sociodemographic factors, but also by modifiable factors, such as smoking habits and breastfeeding difficulties, which can be amenable to public health interventions” [21]. An appreciation of the drivers and patterns of socioeconomic inequalities is vital in designing effective SBC intervention strategies that target population sub-groups most at need, improves its relative impact and in turn, the potential to reduce the inequality gaps.

Estimating the avoidable inequality (proportion of the inequality that is amenable to intervention) [22] is vital in designing SBC intervention strategies and goal setting. Typically, only a proportion of inequalities is amenable to intervention (termed avoidable inequality) [22]. The proportion of the estimated inequality not correctable by interventions (unavoidable inequalities) arises from genetic factors and gender: determinants such that interventions cannot modify. Decomposing inequality estimates into the relative contribution of the associated determinants into the avoidable and unavoidable proportions [22, 23] potentially provides evidence to support better targeting of intervention strategies with more realistic program impact expectations. Although there is increasing worldwide interest in SBC programming to modulate positive behavior and ideational norms, there has not been significant research attention given to examining how inequalities can potentially mitigate the impact of programs, there is limited evidence in the published literature on inequality in the population distribution of priority behaviors and related health-improving ideation factors to inform better targeting of SBC interventions to population groups.

There is sufficient evidence from the published literature to suggest a regressive socioeconomic status or educational level-related inequality in populations [24, 25]. The lower socioeconomic groups are less likely than, the higher socioeconomic groups to acquire positive breastfeeding behavior [25], have limited access to nutritional counselling, and have limited access to factors that generate positive ideation that influence healthy breastfeeding behavior. Although an objective estimation of inequalities in statistically verifiable metrics may provide critical evidence to support the targeting of intervention strategies towards better outcomes and the objective evaluation of such programs and interventions, there has not been sufficient evidence of such explorations in Nigerian populations or elsewhere.

Since 2017, the United States Agency for International Development (USAID) Nigeria has invested in implementing SBC intervention programs through the Breakthrough ACTION Nigeria (BA-N) consortium in 11 of Nigeria’s 37 states.

BA-N aims to increase 17 priority health behaviors in Maternal, Newborn, Child Health plus Nutrition (MNCH+N), Family planning, and Malaria. Its goals are to improve individual and social determinants of health, strengthen SBC coordination and collaboration among partners, and strengthen the SBC capacity of national and sub-national public sector entities. The BA-N SBC intervention program consists of three core components: 1) advocacy outreach to opinion leaders and community influencers at State and LGA levels; 2) direct engagement of community members through household visits and community dialogues directed at target populations, with referrals for services as needed; and 3) complementary integrated SBC messaging through mass, mid-media, and mobile phones. The trust of Breakthrough Action programing and interventions is based on the ideation model of Strategic Communication and Behavior Change.

However, Breakthrough RESEARCH Nigeria (BR-N) is USAID’s flagship project for social and behavior change (SBC) research, evaluation, and generating programmatic evidence to inform and support BA-N’s SBC programs interventions, including other SBC programs in Nigeria in general.

### Objective

This paper seeks to explore the empirical evidence of socioeconomic status (SES) related inequalities in the population distribution of exclusive breastfeeding behavior and practice and the enabling ideation factors in childbearing age women in three north-western Nigerian states of Sokoto Kebbi and Zamfara. The main goal is to objectively estimate the degree of inequality in the practice of exclusive breastfeeding and its association with ideating psychosocial factors by employing the concentration index method [26]. By so doing, the inequality estimate was decomposed to the contributions from the associated ideation factors and sociodemographic determinants. The elasticity of the ideation and sociodemographic determinants with respect to exclusive breastfeeding practices, the avoidable inequality, and the proportion of linear population redistribution of exclusive breastfeeding practices to eliminate the inequality (achieving zero inequality) were estimated.

## Methods

This exploration is based on the household data from a Breakthrough RESEARCH (BR-N) Behavioral Sentinel Surveillance (BSS) baseline survey wave conducted between September and October 2019 in Breakthrough ACTION program areas in Kebbi, Sokoto, and Zamfara.

### Sampling and data

Data for this paper were obtained from a Behavioral Surveillance (BSS) Survey that collected information on several health issues, including breastfeeding practices and factors that might influence these practices. The BSS data were obtained through a representative two-stage cluster-sample, a cross-sectional population survey of over 3000 women with a child under two years in wards from Kebbi, Sokoto, and Zamfara states. The sample is considered representative of the population of women who had a child under two years from in these states. The survey sample size was based on the BR-N evaluation design [27], allowing for a 10% non-response rate, a power criterion of 0.80, an alpha coefficient of 0.05, and varying intra-cluster correlations and minimal detectable differences for priority outcomes of the evaluation. At the first stage, 108 enumeration areas (EA) (36 in each state) were selected from BR program wards using digital maps and a grid sampling methodology. At the second sampling stage, all households within each sampled Enumeration Areas (EA) were randomly sampled to select households with a resident childbearing age (14–49-year-old) woman who had a child under two years. Responses from the sampled women were obtained through face-to-face interviews using the household and female pilot-tested questionnaires by trained interviewers. Information was obtained on usual resident household members and household assets and characteristics. The female questionnaire was used to collect data on respondents’ demographics, reproductive history, contraceptive use, media exposure, gender norms, exclusive breastfeeding and ideations related to breastfeeding while conducting the interviews in the local (Hausa) language.

A currently breastfeeding infant aged 0–5 months, who was neither offered any liquids during the first three days after birth nor any soft or semi-solid foods in the 24 h prior to administering the questionnaire was coded as being exclusively breastfed - the outcome variable (Table 1). A five-item Likert scale response (strongly disagree, somewhat disagree, don’t know, somewhat agree, and strongly agree) was obtained for the ideation questions probing for the respondents’ knowledge and beliefs domain of breastfeeding ideation and norms. Responses were: very uncertain, somewhat uncertain, don’t know, somewhat confident, and very confident for probes into self-efficacy (Table 1). The overall response rate was 99%.

**Table 1:** Descriptive statistics of variables used in the regression

Ideational Dimension	Domain	Questions/probes	Variables	Response category	n	%
			Exclusive breastfeeding six months after birth	Yes	297	9.9
				No	2710	90.1
Cognitive	Knowledge	^a^In your opinion, what are the benefits, if any, for mothers who exclusively breastfeed their infant for the first six months of life?	^a^Spontaneously reports any benefits of EBF practice (first six months of infants’ life) for the mother	YES	1480	49.2
				No	1527	50.8
		^b^What can a mother do to protect the health of her newborn baby immediately after delivery?	Spontaneously mentions immediate breastfeeding as a method to protect the health of the newborn after delivery	YES	1903	63.3
				No	1104	36.7
	Belief	A mother’s breastmilk immediately after birth is bad milk.	Agreed (strongly or somewhat) that mother’s breastmilk after birth is bad milk	Agree	741	24.6
				Disagree	2266	75.4
Social	Injunctive norm	It is important for mothers to only give their child breastmilk during the first 6 months after birth.	Agreed on the importance of mothers to give their child only breastmilk in the first six months of infant’s life	Agree	1760	58.5
				Disagree	1247	41.5
	Descriptive norms	Most women in my community only give their infants breastmilk, and no water, for the first six months after birth	Agreed that most women in the community give infants only breastmilk in the first six months of life.	Agree	1176	39.1
				Disagree	1831	60.9
	Social influence	Besides yourself, who else may influence your decision about whether to breastfeed or not?	Who influences mothers’ decision to breastfeed?	Partner	1444	48.0
				Friend and family or no one else	1563	52.0
			Maternal regular attendance at community meetings	yes	183	6.2
				no	2775	93.8
Emotional	Self-efficacy	How confident are you that you could exclusively breastfeed your child for the first six months of life?	Confident to exclusively breastfeed a child in the first six month	Confident	1500	49.9
				uncertain	1507	50.2
Sociodemographic /Socioeconomic variables.	–		ANC attendance (4+ visits)	yes	705	23.6
				No	2289	76.6
	–		Number of equivalent adults in a household	Mean	3007	2.9
			Maternal Age	15–19	370	12.3
				20–24	895	29.8
				25–29	811	27.0
				30–34	548	18.2
				35–39	268	8.9
				40–44	90	3.0
				45–49	25	0.8
			Maternal education	None or Islamic Primary and higher education	2510 497	83.4 16.5
Socioeconomic status		Household possessions	Household wealth index.	Mean Range Standard deviation	3007	0.369 0–1 0.483

*N* = 3007

^a^ In your opinion, what are the benefits, if any, for mothers who exclusively breastfeed their infant for the first six months of life? Yes responses: “As soon as she thinks she is pregnant”; 15.96%, “In the 1st trimester”, 11.42%; “At the beginning of the 2nd trimester”, 20.49%; “At the beginning of the 3rd trimester”, 9.37%; “Any time during pregnancy”, 15.65%. No responses: “Other”, 1.57%; and “Don’t know”, 25.54%

^b^ What can a mother do to protect the health of her newborn baby immediately after delivery? Reports no benefits or don’t know, 36.7%; Reports any benefit, 63.3%

### Analysis

The concentration index (CIx) [28, 29] method was adopted for the estimation of the SEC inequality. This method is arguably more appropriate than inequality indices derived from social welfare function (defining equity with the social justice approach) [1]. This method allows for objectively computing the inequality metric as it allows for statistical examination of the inequality estimates for precision, allowing for the examination of changes in inequality in a population over time. The Lorenz (concentration) curve, which visually complements the CIx, was used for the visual descriptive examination of the inequalities in exclusive breastfeeding behavior across the three states. Ranking the exclusive breastfeeding practice by household living standard proxied by the household socioeconomic status, beginning from the lowest, the Lorenz (14) curve plots the cumulative proportion of the population against the cumulative proportion of exclusive breastfeeding behavior. The concentration index (CIx) is computed as twice the area between the concentration curve and the diagonal, taking a value of zero if the curve coincides with the diagonal and positive (negative) values when it lies above (below) the diagonal [22]. and is represented in the formula below [30–32]:

1 $$ C=\frac{2}{n\mu}\sum \limits_{i=1}^n{h}_i{R}_i-1 $$

Where *h*_*i*_ is the health sector variable, (exclusive breastfeeding) for person *i*; *μ* is the mean of *h,* and *R*_*i*_ is the fractional rank in the household wealth index distribution of the *i*^th^ person (distribution from most disadvantaged (i.e., poorest) to the least disadvantaged (i.e. richest)) [30–32]. With a negative CIx, inequality is interpreted as pro-poor (favoring the population’s socioeconomic disadvantaged), indicating that individuals with exclusive health practice are disproportionately concentrated among the socioeconomically disadvantaged groups. A positive concentration index implies that the inequality is pro-rich. All the socioeconomic groups enjoy the same distribution of positive breastfeeding behavior, ideational norms, and the enabling factors if the plot coincides with the diagonal (zero inequality). If the inequality estimates favor the population’s socioeconomically advantaged (wealthier) group, multiplying the estimated concentration index by 75 gives the percentage of exclusive breastfeeding behavior that would be needed to be (linearly) redistributed from the wealthier half to the poorer half in the population to arrive at a zero concentration index [33]. The concentration index ranges from − 1 (inequality fully favoring poorer households indicating that all individuals that are exclusively breastfeeding their children are from poorer households and + 1 (inequality favoring wealthier households indicating that all individuals that are exclusively breastfeeding are in the wealthier households) [22, 30–32].

### Decomposition of inequality

We followed Wagstaff et al. (2003) [23] to estimate the overall inequality in the practice of exclusive breastfeeding (*y*_*i*_), decomposing the inequality to the contribution of ideation and sociodemographic determinants (*x*_*k*_) using the convenient linear regression model [23, 30, 32]:

2 $$ {\boldsymbol{y}}_{\boldsymbol{i}}=\boldsymbol{\alpha} +{\sum}_{\boldsymbol{k}}{\boldsymbol{\beta}}_{\boldsymbol{k}}{\boldsymbol{x}}_{\boldsymbol{k}\boldsymbol{i}}+{\boldsymbol{\varepsilon}}_{\boldsymbol{i}} $$

The concentration index is computed following Wagstaff et al., [29] and Kakwani et al. [30], *C* can be computed alternatively as:

3 $$ \boldsymbol{C}={\sum}_{\boldsymbol{k}}\left({\boldsymbol{\beta}}_{\boldsymbol{k}}{\bar {\boldsymbol{x}}}_{\boldsymbol{k}}/\boldsymbol{\mu} \right){\boldsymbol{C}}_{\boldsymbol{k}}+\boldsymbol{G}{\boldsymbol{C}}_{\boldsymbol{\varepsilon}}/\boldsymbol{\mu} $$

Where μ is the mean of *y*_*i*_ as previously defined (eq. 1), ${\overline{x}}_k$ the mean of *x*_*k*_, *C*_*k*_ is the concentration index for *x*_*k*_, *k* is the vector of variables and *GC*_*ε*_ is the generalized concentration index for *ε*_*i*_. The deterministic or explained component (the first component in eq. (2)) is equal to the weighted sum of the concentration indexes of the regressors where weights are the elasticities of *y*_*i*_ with respect to *x*_*k*_. The second component (computed as a residual) is the unexplained component, reflecting the inequality in ideation that cannot be explained by systematic variation in *x*_*k*_ across socioeconomic groups. This decomposition allows further decomposition of each factor’s contribution to the elasticities of the breastfeeding practices $\left({\beta}_k{\overline{x}}_k/\mu \right)$ and SES-related inequity (*C*_*k*_) [32]. Standardization was done to control for possible confounding effects from sociodemographic variables [30, 34–37], and to estimate the difference between the observed and actual inequality, including the degree of inequality that should be observed if the standardizing variables were uniformly distributed across the population (which by extension, purges the effects of confounders revealing the potentially avoidable inequality). Avoidable inequality is defined as the level of inequality that can be ameliorated through interventions [22, 34, 36, 37]. Inequalities arising from genetic, regional, and temporal factors may be impossible to change. We used this convenient (multivariate) regression method to decompose the estimated inequality into 1) the contribution from ideational and normative determinants, 2) the innate inequality in these determinants, and 3) the elasticities of the determinants on the inequality in exclusive breastfeeding behavior [22, 23, 30]. Also, estimates of the avoidable inequality (the proportion of the inequality that is amenable to intervention or programming) and unavoidable (not amenable to programming and interventions) components of the total decomposed inequality were obtained. The relative contribution of the sociodemographic determinants of inequality, such as respondents’ age, educational status, household size (measured in adult equivalence), and other determinants of interest, were estimated. Each household size was standardized using the household adult equivalence (an equivalent number of all household members). The household adult equivalence estimated using the formula - AE = (A + αK)^θ^ [38, 39] (AE = adult equivalence, K = number of under 18 years old in a household). Deaton and Zaidi (2002) propose values in the region of 0.3 to 0.5 for α (higher in developed countries) and 0.75 to 1.0 for θ, given that food accounts for a large proportion of total consumption and economies of scale are relatively limited [39, 40].

The unit of analysis was the household or individuals within the households ranked by the socioeconomic status (SES) or household living standard. Socioeconomic status was proxied by household wealth index estimated through principal component analysis of household possessions (assets) [22, 41–44]. The analysis was based on the bivariate approach to measuring inequalities [26, 45]. This approach looks at the subset of breastfeeding behavior and ideation factors inequality occurring across the distribution of households or individuals (ranked by SES) by typically comparing the cumulative proportion of households or individuals ranked by ideation or SBC behavior against the cumulative proportion of SES [28, 46–49]. The five-scale Likert responses were dichotomized to fit the bivariate regression model for estimating the Lorenz curve and the CIx index. All the “strongly” or “somewhat agree” responses were recategorized as “yes” or “agreed” while all other responses including “strongly” or “somewhat disagree” and “don’t know”, were classed as “no” that is, not explicitly agreeing. All correct responses to the question probing the knowledge of the respondents: “In your opinion, what are the benefits, if any, for mothers who exclusively breastfeed their infant for the first six months of life?” were coded as “yes” for spontaneously reporting any benefits of EBF practice (first six months of infants’ life) Table 1. The “don’t know” responses and were coded as “no”.

**Fig. 1:**
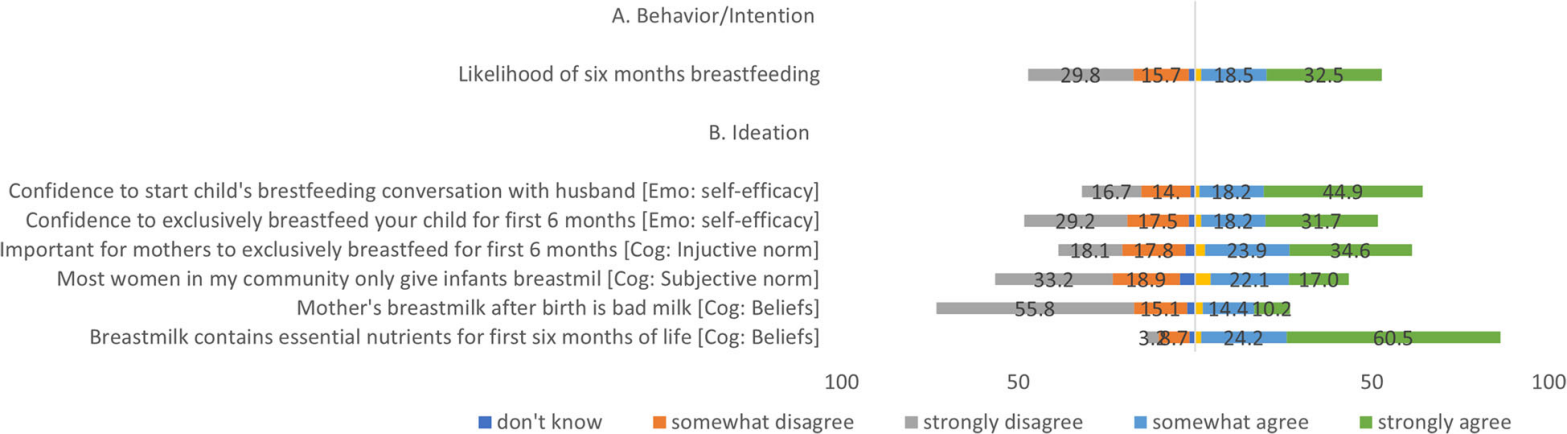
Diverging bar Description of Exclusive breastfeeding behavior and ideation factors. Likert scale responses to exclusive breastfeeding behavior/intention and ideation queries positively skewed towards (somewhat and strongly agreeing with the behavior and ideation probes

The Lorenz estimate, Lorenz curve, and the Foter-Greer-Thorbecke (FGT_CI) routines of STATA 16© (STATA Corporation, College Station, TX, USA) statistical software was used for all analyses. Also, Microsoft® Excel spreadsheet was used to construct the diverging bar graph (Fig. 1). We used a two-part (Probit and generalized linear model) multiple regression model to adjust for the excess zeros in the exclusive breastfeeding outcome variable in the data. Akaike Information Criteria (AIC) was used for diagnostics and selecting the best model.

## Results

### Descriptive statistics for the sample

Table 1 shows the summary description of the full sample. A total of 3007 childbearing age women were included in the analysis. Among the study participants, about 10% had practiced exclusive breastfeeding after birth. About 30% of the women were in the modal 20–24 years age bracket, 23.6% had at least four antenatal care visits during her last pregnancy, majority (83%) had no education or had Islamic education, and only 6.2% regularly attended community meetings in the past year. The mean number of equivalent adults per household was about 3. Regarding the ideation factors in the cognitive dimension, half (49.2%) of the women spontaneously mentioned any benefit from exclusive breastfeeding in the first six months of a child’s life (knowledge domain). About 63% spontaneously mentioned immediate breastfeeding after birth as a method to protect the newborn’s health (in the knowledge domain), and 70.9% did not agree with the belief that the mothers’ breastmilk after birth (*colostr*um) is bad milk (in the belief domain). On the social ideational dimension, 58.5% of the participants agreed that it is important for mothers to give their child only breastmilk in the first six months of infant’s life (Injunctive norm); 39.1% agreed that most women in the community give infants only breastmilk in the first six months of life (descriptive norm); and 48% mentioned their partners as influencing their decision to breastfeed (social influence). For the emotional dimension, 49.9% of the participants stated their confidence in exclusively breastfeeding a child in the first 6 months after birth (self-efficacy).

The diverging bars in Fig. 1 describe the Likert scale responses to the probes on exclusive breastfeeding behavior and the associated ideational factors. About 32.5 and 18.5% strongly or somewhat agreed, respectively, regarding their likelihood to breastfeed their next child for 6 months after birth, while 29.8 and 15.7% strongly and somewhat disagreed, respectively. Approximately 44.9 and 18.2% strongly or somewhat agreed, respectively, to have the confidence to start a breastfeeding conversation with their husbands (self-efficiency in the decision-making domain in the emotional dimension) and lower percentages, 16.7 and 14.0% respectively, disagreed. Also, within this domain and dimension, 31.7 and 18.2% strongly or somewhat agreed, respectively, to have the confidence to exclusively breastfeed the child for the first 6 months of birth, 29.2% strongly disagree, and 17.5% somewhat disagreed. Regarding the cognitive dimensions: 34.6% strongly agree, 23.9% somewhat agree, while 18.1% strongly disagree, 17.8% somewhat disagree that it is essential for mothers to exclusively breastfeed for 6 months (injunctive norm). About 17.0% of the mothers strongly agree, and 22.1% somewhat agree that most mothers in the community only give infants breastmilk (subjective norm) while 33.2 and 18.9% strongly and somewhat agree, respectively. Approximately 10.2% strongly agreed, and 14.4% somewhat agree that a mother’s breastmilk after birth is bad (belief domain) while 55.8% strongly disagree, 15.1% somewhat disagree. Finally, 60.5% strongly agree, and 24.2% somewhat agree that breastmilk contains essential nutrients for 6 months of life (beliefs), 8.7% strongly and 3.2% somewhat disagree.

### Lorenz (concentration) curve

**Fig. 2:**
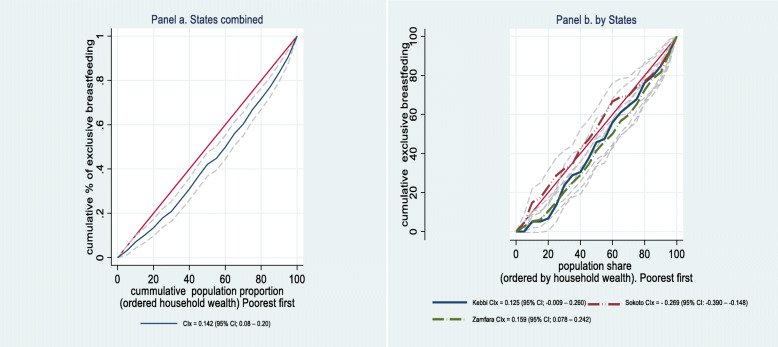
Lorenz (Concentration) Curves: Inequality in Exclusive breastfeeding practice among women in Sokoto Kebbi and Zamfara states

Figure 2 shows the Lorenz concentration index for exclusive breastfeeding behavior in the three states combined data (panel a) and by state (panel b). Inequality in exclusively breastfeeding generally favors the socioeconomically advantaged (pro-rich) (CIx = 0.142 (95% CI; 0.08–0.20)). Women who practice exclusive breastfeeding in all three states are disproportionately concentrated in socioeconomically advantaged households, (Fig. 2, panel a). Similarly, in Zamfara and Kebbi states, inequality in exclusively breastfeeding favors the socioeconomically advantaged (CIx = 0.159 (95% CI; 0.078–0.242) and 0.125 (95% CI; − 0.009 – 0.260) respectively), although CIx is not strongly statistically significant. The women who practice exclusive breastfeeding are disproportionately concentrated in socioeconomically advantaged households, (Fig. 2, panel b). In contrast, the inequality in exclusively breastfeeding children is pro-poor, favoring the socioeconomically disadvantaged in Sokoto State (CIx = − 0.269 (95% CI: − 0.390 – − 0.148)), statistically significant and significantly different from the estimates of Kebbi and Zamfara states. Women who practice exclusive breastfeeding in Sokoto State are disproportionately concentrated in socioeconomically disadvantaged households.

### Generalized linear model analyses

The two-part regression results examining the relationships between the exclusive breastfeeding behavior and the ideation factors and sociodemographic determinants are presented in Table 2. The findings suggest that four or more antenatal clinic attendance during pregnancy significantly accounts for the variation in exclusive breastfeeding. Ideation factors: influence of partners in the decision to breastfeed the child and spontaneously mention any benefits of EBF practice (first 6 months of infants’ life) for the mother, also are significantly associated with the exclusive practice of exclusive breastfeeding. The best fitted regression results from the FGT_CI routines excludes the sociodemographic variables: the number of equivalent adults in a household, maternal age and maternal education.

**Table 2:** Generalized linear regression results for Exclusive breastfeeding behavior

	Coef.	Robust Standard Error	z	P > z	[95% Confidence Interval]
**Spontaneously reports any benefits of EBF practice (first six months of infants’ life) for the mother**	− 0.0007	0.0002	−3.64	0.00**	−0.0010 - -0.0003
**Spontaneously mentions immediate breastfeeding as a method to protect the health of the newborn after delivery**	−0.0001	0.0003	−0.44	0.66	−0.0007 - 0.0004
**Agreed (strongly or somewhat) that mother’s breastmilk after birth is bad milk**	0.0001	0.0005	0.27	0.79	−0.0009 - 0.0012
**Agreed on the importance of mothers to give their child only breastmilk in the first six months of infant’s life**	0.0001	0.0005	0.31	0.76	−0.0008 - 0.0010
**Confident to exclusively breastfeed a child in the first six month**	−0.0002	0.0004	−0.47	0.64	−0.0010 - 0.0006
**ANC attendance (4+ visit)**	0.0624	0.0214	2.92	0.00**	0.0205–0.1044
**Number of equivalent adults in household**	−0.0570	0.0308	−1.85	0.06	−0.1174 - 0.0033
**Maternal age**	0.0023	0.0063	0.36	0.72	−0.0101 - 0.0147
**Agreed that most women in the community give infants only breastmilk in the first six months of life**	0.0003	0.0003	1.17	0.24	−0.0002 - 0.0009
**Maternal regular attendance at community meetings**	0.0242	0.0407	0.6	0.55	−0.0556 - 0.1040
**Who influences mothers’ decision to breastfeed?**	0.0424	0.0169	2.51	0.01**	0.0093–0.0755
_cons	0.1403	0.0326	4.3	0.00	0.0764–0.2042

**Pvalue = 0.000, Log pseudolikelihood = −33,293.97, Deviance = 3517.69, (1/df) Deviance = 1.1989, Pearson = 3517.69, (1/df) Pearson = 1.19894, AIC = 22.61, Variance function: V(u) = 1 [Gaussian], Link function: g(u) = u [Identity]

### Concentration index and decomposition analysis for inequality of exclusive breastfeeding practices

Table 3 presents the CIx analysis results of the decomposition of the CIx into the contribution of the ideation and sociodemographic variables the estimated inequality (in the fourth column); the elasticities of the variables with respect to the exclusive breastfeeding behavior (third column); and the inequalities in these factors (second column). Inequality in exclusive breastfeeding in the three states combined was pro-rich as mothers in wealthier households were more likely to engage in exclusive breastfeeding (CIx = 0.1236; *p*-value = 0.00). ANC attendance 4+ was the most significant contributor to the inequality (CIx = 0.0307; *p*-value = 0.00). The contributors to inequalities in exclusive breastfeeding practices were mainly in the cognitive and emotional dimensions, although their contributions to the inequality were not statistically significant (Table 3).

**Table 3:** Decomposition of household inequality Exclusive Breastfeeding behavior

Ideation factors	Concentration indexes	Elasticities	Contributions
Spontaneously reports any benefits of EBF practice (first six months of infants’ life) for the mother	−0.1986**	− 0.3289	0.0653
Spontaneously mentions immediate breastfeeding as a method to protect the health of the newborn after delivery	−0.2443**	−0.0262	0.0064
Agreed (strongly or somewhat) that mother’s breastmilk after birth is bad milk	−0.3935**	0.0055	−0.0022
Agreed on the importance of mothers to give their child only breastmilk in the first six months of infant’s life	−0.2507**	0.0089	−0.0022
Confident to exclusively breastfeed a child in the first six month	0.3463	0.0078	0.0027
ANC attendance (4+ times)	0.3722**	0.0825**	0.0307**
Maternal age	0.0161**	0.0674	0.0011
Agreed (strongly or somewhat) that most women in the community give infants only breastmilk in the first six months of life	−0.2365**	0.0265	− 0.0063
Maternal regular attendance at community meetings	0.1887**	0.0100	0.0019
Who influences mothers’ decision to breastfeed?	0.0042	0.1484**	0.0006
Residual			0.0250
**Total**			0.1236**

** *P* value = 0.000. The estimates are significantly nonzero

### Elasticities

The elasticity estimates showed that a percentage change in the ideation factor of partners influencing the decision to breastfeed and attending ANC 4 or more times (ANC 4+) during the last pregnancy could significantly result in a 0.15% (*p*-value = 0.00) and 0.08% (p-value = 0.00) change in the exclusive breastfeeding behavior respectively. The estimates of the elasticity of exclusive breastfeeding relative to the other ideation factors were not statistically significant.

### Inequalities in the ideation determinants

The results showed that there are inequalities in the ideation determinants in themselves. Inequalities in a few of the determinants are pro-rich while majority are pro-poor (Table 3). The inequality in the regular attendance at community meetings (CIx = 0.189; *p*-value =0.00); ANC4+ (CIx = 0.372; p-value = 0.00); and maternal age (CIx = 0.016; p-value = 0.00), were significantly pro-rich. Inequalities in the majority of the ideation determinants are pro-poor, including spontaneously reporting any benefits in the first six months of exclusive breastfeeding practice (CIx = − 0.1986, p-value = 0.00); Spontaneously mentioning immediate breastfeeding as a method to protect the health of the new-born after delivery (CIx = − 0.2443, p-value = 0.00); Agreeing that mothers breastmilk after birth (colostrum), is bad milk (CIx = − 0.3935, *P*-value = 0.00); Agreeing on the importance of mothers to give their child only breastmilk in the first six months of infant’s life (CIx = − 0.2507, p-value = 0.00); Confidence in practicing EBF for first six months of infant’s life (CIx = − 0.3463, p-value = 0.00); Agreeing (strongly or somewhat) that most women in the community give infants only breastmilk in first six months of life (CIx = − 0.2365, p-value = 0.00) were pro-poor, that is, the inequalities favor the poorer households.

The results showed that all (100%) the inequality in the distribution of exclusive breastfeeding behavior is avoidable. Also, 11% linear population redistribution of exclusive breastfeeding behavior from the wealthier half to the poorer half of the population could eliminate the inequality in exclusive breastfeeding (CIx = 0, the inequality lying on the line of zero inequality).

## Discussion

This paper assesses the inequality of exclusive breastfeeding practice and the contribution of ideational and sociodemographic determinants in three north-western (Sokoto, Kebbi, and Zamfara) states of Nigeria. The analyses provide evidence of inequality in exclusive breastfeeding practice and its SBC ideational determinants of the practice of exclusive breastfeeding that interventions may target for optimal program impact. For instance, we found that the inequality of exclusive breastfeeding generally disfavors the socioeconomically disadvantaged – pro-rich. Women in socioeconomically disadvantaged households were less likely than their counterparts in socioeconomically advantaged households to practice exclusive breastfeeding. Four or more antenatal visits during the last pregnancy, the influence of partners in the decision to breastfeed the child in the social-influence communication domain, and spontaneously reporting any benefits of EBF practice in the first six months of infants’ life in the knowledge domain are significantly associated with the practice of exclusive breastfeeding. The women in the wealthiest households were more likely to exclusively breastfeed the child than those in the lower socioeconomic (poorer) households. Regular antenatal (at least four) visits during pregnancy, which is also pro-rich, contributed significantly to breastfeeding practices’ inequality. The findings showed that changes in the population distribution of women who had at least four antenatal visits during pregnancy, women whose partners influenced their decision to breastfeed the child exclusively and knew benefits from exclusive breastfeeding for the child significantly changed the practice of exclusive breastfeeding. The results could reflect the fact that better-off individuals might have better access to the antenatal clinic during pregnancy, where exclusive breastfeeding is a regular topic during health education sessions and is commonly a part of advocacy messages.

These results have implications for SBC programming in northern Nigeria, where the poorer, socioeconomically disadvantaged groups are consistently disadvantaged in the population’s access to healthcare [50, 51]. For instance, SBC interventions often employ multichannel approaches, including the use of broadcasting mass communication approaches (using the mass media) for behavioral change, as it is in the three north-western states of Nigeria. An implicit assumption in such intervention strategy and the expected impact is that all population groups are uniformly exposed to the intervention and impacted is assumed to be uniform across all strata. Non-uniform (inequal) exposure to population-based SBC interventions could generate a skewed, non-uniform response across population sub-groups. This can potentially minimize the intensity of program impact in the disadvantaged groups and favoring the advantaged groups in the population and ultimately resulting in suboptimal, inefficient production of program impact.

This tendency for inefficient production of impact is directly proportional to the degree of the inequality as captured in the estimates of the concentration indexes. The more the inequality in the breastfeeding behavior, as the estimate of the concentration index tend towards + 1, the less likely will the population of women in households in the lower socioeconomic strata, be exposed to doses of interventions comparable to the women in the socioeconomically advantage strata. Ultimately, the interventions are unlikely to impact all the socioeconomic strata of the population equally but to the disadvantage of the socioeconomically disadvantaged This study showed a disproportionate population distribution of the endowment of exclusive breastfeeding and the enabling ideation factors to the disadvantage of the women with under-two-year-old children in poorer subgroups in the population. It can be similarly argued that the marginal propensity for program or intervention impact is higher among the subgroups with the least distribution of exclusive breastfeeding behavior (women with under-two-year-old children from the lower socioeconomic groups of the population) than in subgroups with saturated (also, in this case, are those in higher socioeconomic groups). SBC programs and interventions for improving populations’ exclusive breastfeeding behavior could efficiently achieve a more optimal impact if the inequality in exclusive breastfeeding behavior was addressed during intervention. The population subgroups with higher risk (poorer exclusive breastfeeding behavior) and potentially limited access (socioeconomically disadvantaged) require more intense intervention than the advantaged subgroups in the population to potentially achieve more SBC program impact. SBC communications for improving positive breastfeeding behavior could explore strategies for deliberate targeting or intensifying programming among the disadvantaged.

The findings suggest that improving and expanding ANC coverage through pro-poor intervention could improve breastfeeding practices among the socioeconomically disadvantaged group of the population. The results showed that inequality in exclusive breastfeeding practice is totally (100%) amenable and could be mitigated through targeted interventions. Sociodemographic factors, which are unmodifiable (constants) were not significant contributors to the inequality in exclusive breastfeeding. Programmatically achieving a reduction in the inequality in exclusive breastfeeding behavior by 10% could eliminate the inequality (zero inequality) in the population distribution of positive exclusive breastfeeding behavior.

The elasticity estimates are measures of the responsiveness of exclusive breastfeeding behavior in response to a change in the ideational factors (independent variables). It provides an estimate of the extent to which programmatic improvement in n ideation factor may result in a change in breastfeeding behavior. A percentage increase in the rate of ANC 4+ visits will result in a 0.083% change in exclusive breastfeeding behavior. Messages that advocate and reinforce exclusive breastfeeding behavior, positively modulating ideation and breastfeeding norms are actively provided in the health education routines for pregnant women attending ANC clinics in primary care clinics in Nigeria. The results showed inequality in antenatal care visits (ANC 4+ CIx of 0.3722) favors the women with under-two-year-old children from socioeconomically advantaged households. That is, women from richer households attend antenatal clinics during pregnancy, and are more exposed to the positive breastfeeding behavior reinforcing messages than those in disadvantaged (poorer households in the study population.

Also, the elasticity of spousal communication, in terms of a woman’s partner influencing her decision to breastfeed the child significantly motivates positive breastfeeding behavior in the women. A percentage change in this variable results in a 0.14% change in exclusive breastfeeding practice. This suggests the importance of male roles in influencing the decision to breastfeed the child and programmatic enhancement of could be effective in improving population breastfeeding behavior. Further exploration into the role of the males in SBC communication and in the decision to exclusively breastfeed the child is necessary for informing future interventions.

In addition to the inequality in ANC 4+, there were inequalities also, in the ideational factors independently in themselves. For instance, spontaneously reporting any benefits of EBF; spontaneously mentioning immediate breastfeeding as a method to protect the health of the newborn after delivery; and agreeing (or disagreeing) that a mother’s breastmilk after birth (colostrum) is bad milk were disproportionately concentrated among the socioeconomically disadvantaged households - pro-poor. Similarly, agreeing on the importance of mothers to give their child only breastmilk in the first six months of infant’s life; and agreeing that most women in the community give infants only breastmilk in first six months of life, are pro poor. Notably, these are in the knowledge domain of the cognitive dimension and the injunctive and descriptive domains of the social dimension of the SBC and Kincaid communication models. Maternal regular attendance at community meetings (in the social-influence domain of the social dimension of communication models) are disproportionately concentrated in the socioeconomically advantaged households – pro-rich. We observe that exclusive breastfeeding practices are mainly influenced by ideational domains within the cognitive and social dimensions of the SBC communication models, unlike those within the emotional dimension. Ideations in these dimensions could be the strategic focus of SBC programming and intervention strategies.

A similar exploration of inequality in exclusive breastfeeding behavior and enabling ideating norms is scarce in the published literature [52]. A systematic review did not reveal similar studies that explored inequality in exclusive breastfeeding, whose results could be compared with the results of this study. However, the results from this study provide evidence and the basis for future comparison of the evidence of inequality in exclusive breastfeeding practice. The results of this studies could be valid for the states in the northwest and other regions in northern Nigeria with comparable sociodemographic and sociocultural settings. The northwest region of Nigeria has the highest poverty rates relative to the southern regions. Traditional health practices especially around pregnancy and childbirth remain culturally preferred and accepted than orthodox health care. These circumstances explain the findings in this study around the inequality in antenatal care visits during pregnancy disfavoring women with under-two-year-old children from the poorer households. These women are confronted with limited access to primary health care are less exposed to breastfeeding behavior reinforcing health education messages, compared to the women from richer households. Further studies are necessary to advance the understanding of the inequality in exclusive breastfeeding practice and its ideating factors.

### Limitations

This study has three notable limitations. First, data from a cross-sectional population survey restricts the inferences around causal relationships between the exclusive breastfeeding practice and the ideational determinants. Second, likely that the self-reported (self-assessed) responses for breastfeeding behavior could have recall bias with a possible underestimation that may affect the magnitude of the associations analyzed in the CIx and decomposition models. Individuals in lower socioeconomic groups have tended to rate their health more optimistically than those in higher socioeconomic groups [53, 54]. The socioeconomically advantaged mothers may report better self-rating [53, 55]. The analysis did not account for the possible response bias. Thirdly, the Likert scale response is problematic for its possibility of responses (due to social desirability), fatigue/inattention, and subjective interpretation biases. Also, collapsing the Likert scale variables to bivariate variables may have introduced categorization biases also.

## Conclusions

Inequalities in the population distribution of exclusive breastfeeding practice, a priority SBC behavior, and its associated ideational determinants exist among women of childbearing age with a child under two in the north-western states Nigeria disfavoring the socioeconomically disadvantaged in the population. The inequality is mostly avoidable and is amenable to programmatic intervention. A 10.7% redistribution could eliminate the inequality. Messaging and communications for SBC programs and interventions to improve breastfeeding practices could be more effective by targeting the mitigation of these inequalities among the population’s socioeconomically disadvantaged groups. Antenatal care visits during pregnancy and spousal communication (male role) are important influencers of positive breastfeeding behaviors. This study contributes to the evidence of inequality in exclusive breastfeeding practice with objective metrics. Further studies would benefit from contrasting the results of this study with studies in other regions or across other health areas and exploring the male (spouse) role in SBC breastfeeding practice.

## Data Availability

The data that supports the findings of the current study are available from the corresponding author upon reasonable request.

## Abbreviations

AIC: Akaike Information Criteria
ANC4+: Four or more antenatal clinic visits during a pregnancy
BA-N: Breakthrough ACTION, Nigeria
BR-N: Breakthrough RESEARCH, Nigeria
BSS: Behavioral Sentinel Surveillance
CI: Confidence interval
CIx: Concentration Index
EA: Enumeration Area
EBF: Exclusive breastfeeding
FGT-CI: Foter-Greer-Thorbecke - Concentration Index
FP: Family Planning
LGA: Local Government Area
MNCH + N: Maternal, Newborn and Child Health plus Nutrition
SBC: Social and Behavioral Change
USAID: United States Agency for International Development
